# Supplementary material for: Comparative multiomics analysis of cell physiological state after culture in a basket bioreactor
Source: Sci Rep. 2022 Nov 23;12:20161. doi: 10.1038/s41598-022-24687-4 (PMC9686226; doi:10.1038/s41598-022-24687-4)
Supplement: Supplementary file 1 — Supplementary Information 1. [file 41598_2022_24687_MOESM1_ESM.zip › raw data/Transcriptomic raw data/6.Enrichment/2.KEGG/TE1vsTC1/ALL/src/csab04810.html]

csab04810


- csab:103240591

- Up regulated genes

103240591(1.99059850624449)

- csab:103224044

- Up regulated genes

103224044(1.13120552242456)

- csab:103224044

- Up regulated genes

103224044(1.13120552242456)

- csab:103223856

- Down regulated genes

103223856(-1.2335633691695)

- csab:103224900

- Down regulated genes

103224900(-1.42998371610984)

- csab:103231401

- Down regulated genes

103231401(-2.56891951280362)
- csab:103243277

- Up regulated genes

103243277(1.88743299439371)

- csab:103246479

- Down regulated genes

103246479(-1.5490263767958)

Close
